# Do coaching style and game circumstances predict athletes' perceived justice of their coach? A longitudinal study in elite handball and volleyball teams

**DOI:** 10.1371/journal.pone.0205559

**Published:** 2018-10-15

**Authors:** Maarten De Backer, Bart Reynders, Filip Boen, Stef Van Puyenbroeck, Gert Vande Broek

**Affiliations:** KU Leuven, Faculty of Kinesiology and Rehabilitation Sciences, Department of Kinesiology, Leuven, Vlaams-Brabant, Belgium; Universitat de Valencia, SPAIN

## Abstract

**Objective:**

The present longitudinal study is the first to examine game to game fluctuations of perceived justice of elite volleyball and handball coaches. More specifically, we studied whether coaching style (i.e., need support versus control), coach behaviors (decision justifications), player’s status (i.e., starter or substitute), and game result (win/loss) predicted athletes’ perceived justice and its fluctuations.

**Methods:**

A longitudinal questionnaire study was performed during 6 consecutive weeks among Belgian female volleyball (*N* = 57) and male handball players (*N* = 39). We administered a general questionnaire (i.e., need support/control) the first week, and game-specific questionnaires (i.e., justice, decision justifications, game circumstances) after six consecutive games. Because game-to-game measures (i.e., within-athlete) were nested into individuals (between-athletes) we conducted Hierarchical Linear Modeling to examine the hypotheses.

**Results:**

Multilevel analyses showed that 49% of the variance of perceived justice was situated at the within-athlete level. Furthermore, coaches’ need support and the provision of decision justifications were positive predictors of athletes’ perceived justice of the coach. More specific, the impact of justifications was less strong in a high need supportive environment and stronger in a high controlling environment. Finally, both the status of the player and the game result were negative predictors of athletes’ perceived justice.

**Conclusions:**

We can conclude that athletes’ perceived justice of their coach shifts considerably from game-to-game. Furthermore, the coaching style and coaching behaviors can help to overcome the negative effects of specific game circumstances such as being a substitute or losing a game on athletes’ perceived justice of the coach.

## Introduction

When a team is performing well, coaches are often praised for their work. However, in an elite sport setting, a losing streak may quickly result in a crisis in which the coaches’ work is often the first to be doubted (e.g., José Mourinho in his second tenure at Chelsea Football Club [1]). Besides losing streaks, an additional risk for the functioning of sport teams are dissatisfied substitutes who feel unfairly treated by their coach. For example, Bojan Krkic, who was believed to be the greatest talent at FC Barcelona since Lionel Messi, was dissatisfied with his playing minutes and stated on his departure: “Guardiola (i.e., former head coach) was not fair with me on several occasions, and this is one of the reasons that I decided to leave” [2]. Recent studies support this assumed relationship between athletes’ perceived justice of their coach and the quality of athletes’ functioning. For example, it has been indicated that the extent to which athletes feel treated in a fair manner positively predicts: (a) their commitment to the team [3], (b) group dynamic processes such as team identification, cohesion, and social loafing [4,5], and (c) the level of athletes’ self-reported progression[6]. Taking into account the importance of perceived justice, the current study aimed to explore how justice unfolds over time. Moreover, we studied whether the coaching style, the result of the game and the status of the athletes (i.e., starting team or substitute) accounted for between- and within- person variance of players’ perceived justice of the coach.

To our knowledge only Holtz and Harold [7] have performed a longitudinal examination of perceived justice. In line with the statement of George and Jones[8] that organizational phenomena are inherently dynamic and are expected to change over time, Holtz and Harold [7] showed that perceived justice fluctuates over time. However, their findings are inconsistent with the Uncertainty Management Theory (UMT [9–10]). According to UMT, subordinates quickly form overall justice perceptions in order to reduce uncertainty and to help guide subsequent attitudes and behavior. Once the justice perceptions have been formed change is assumed to occur only in the face of relatively dramatic events, such as changes in leadership or organizational restructuring [9]. However, this assumption of perceptual stability in justice perceptions runs counter to the view that virtually all organizational phenomena change over time [8] and has not yet been empirically tested. Consequently, until today it remains unclear whether and how justice perceptions unfold over time and conflicting perspectives regarding the (in)stability of justice persist.

Competitions between elite sport teams constitute very dynamic settings, in which players and coaches have to deal with continuously changing circumstances and weekly public performance evaluations. A longitudinal study in this specific microcosm provides a unique opportunity to address the limited number of studies examining the dynamical characteristics of organizational justice. Therefore, the first aim of the present study was to focus on change in justice perceptions in order to unravel variability that cross-sectional research cannot detect. In addition, the longitudinal perspective makes it possible to check to what extent both the coaching style and the fluctuations in game circumstances (i.e., winning/losing and starter/substitute) are linked to athletes’ perceived justice of the coach.

In order to examine athletes’ perceived justice of the coach and its predictors, we draw upon two highly recognized theoretical frameworks, the Organizational Justice Theory (OJT [11]) and the Self-Determination Theory (SDT [12]). Perceived justice is a concept derived from the OJT, which was originally conceptualized “to describe and explain the role of fairness as a consideration in the workplace” [13] (p 400). In the present study, we decided to focus on distributive and procedural justice. Distributive justice refers to how fair outcomes were distributed among subordinates [13]. Typical outcomes related to distributive justice in a team sport context are the playing time or the assigned position of an athlete. By contrast, procedural justice deals with the fairness of the procedures used to determine these outcome distributions [13]. In sport teams an athlete can be dissatisfied with his status as substitute when he/she perceives the procedures used to determine this status as unfair (e.g., inconsistent, inaccurate). However, if he/she perceives the procedures used to select the starting team as fair (e.g., the coach uses objective scouting information), the athlete is more likely to accept the final decision [14].

Holtz and Harold [7] showed that the within person variance accounted for 29% of the total variance of subordinates’ overall perceived justice of their supervisor in the business setting. These results are in line with previous research on change in other attitudinal constructs (e.g., satisfaction or commitment). A review of the attitude change literature by Lord et al. (p. 734) [15] indicated that “attitude reports can change from one time to the next without any additional information about the attitude object, simply because the context of the moment makes a biased subset of knowledge about that topic cognitively accessible”. Consequently, we assumed that overall justice perceptions will fluctuate considerably over time in a team sport context.

### General predictors of perceived justice: Coaching style

In addition to detecting weekly fluctuations in athletes’ perceptions of justice, we also examined to what extent coaches and game circumstances predict these weekly fluctuations. First, we aimed to examine the impact of the general coaching style. Therefore, we studied the effect of a need supportive and a controlling coaching style. Both leadership styles are rooted in the Self-Determination Theory [12] which has received increasing attention in the domain of sport psychology and coaching. SDT assumes that people have three innate psychological needs (i.e., autonomy, competence, and relatedness), which have to be fulfilled in order to foster growth, integrity and well-being[12]. The need for autonomy implies that people have a natural desire to have a sense of volition and perceive themselves as the origin of their behavior [13]. The need for relatedness implies that people want to feel connected to and respected by others [13]. The need for competence refers to the longing to feel effective and skillful in the activities that one undertakes [13, 16]. Coaches whose behavior is directed towards collectively supporting these three needs are therefore considered as need supportive coaches [17]. To support the three psychological needs of athletes, coaches may demonstrate an interest in athletes’ input or initiative, provide explanatory rationales, recognize athletes’ feelings and perspectives, acknowledge and accept athletes’ negative affect, and avoid overt control [18, 19]. Furthermore, coaches can show concern about the players’ wellbeing, on and off the field [20]. Finally, coaches can provide informational feedback, express their confidence in players, structure expectations, and provide help when needed [21].

Despite the possible beneficial effects of such an approach, coaches’ personality characteristics, the team dynamics and the specific sport context could direct coaches towards a more directive, controlling coaching style [21]. To avoid any misunderstanding, we like to emphasize that in the current manuscript the concept of control refers to psychological control as described within the Self-determination Theory [22]. Controlling coaches impose the way they expect that players behave, think and feel according to their perspective [23]. Strategies such as negative conditional regard, coercive demands, intimidation, guilt induction, and neglecting explanatory rationales, are examples of a controlling coach behavior [22]. As a result, athletes will feel like there is little room to express their own opinions [24].

Research linking need support and psychological control to perceived justice is scarce. However, three managerial strategies (i.e., providing voice, managerial account and consideration) have been shown to increase employees’ perception of justice. These three strategies are very (dis)similar to the concepts of need support and control. First, *voice* which has been defined as the opportunity to express one’s views before a decision is made, shows similarities with autonomy-supportive strategies such as letting room for athletes’ input or initiative, and recognizing athletes’ feelings and perspectives [25]. In contrast, a ‘*no-voice’ condition* shows similarities to a controlling coaching style. In the justice literature no-voice conditions are described as silence or mute conditions, in which people are not involved in the decision making process, nor get the opportunity to express their opinions, or to take initiative [26]. People who are silenced reported lower levels of perceived justice [27]. The second strategy, *managerial accounts* [26], refers to the provision of causal information about the motives of a decision maker's behavior. Such a specific and sincere rationale not only specifies necessary information, but also makes employees feel treated in a respectful and fair manner [28]. The provision of meaningful rationales and informational feedback seem to resemble a need supportive coaching style, whereas it differs from controlling coaching strategies such as neglecting explanatory rationales and coercive demands. The third strategy, showing *consideration* [14], implies that managers support their employees and treat them with dignity and respect. Again, this strategy shows strong similarities with a need supportive coaching style. Both concepts refer to authorities who are concerned with their subordinates and who support them to ensure that they feel respected. In contrast, controlling coaches’ negative conditional regard may result in players who feel less appreciated, or respected than others.

In addition to the clear link with justice-enhancing strategies from the business setting, research has also demonstrated that a need supportive environment increases players’ tactical awareness [29]. This is a logical consequence of the fact that need supportive coaches do allow their athletes to express their views and offer them relevant information about decisions. Consequently, athletes gain an increased knowledge and a better understanding of the decisions made by the coach. Players and coaches are on the same page and will share a clear framework resulting in more predictable decisions which are supported and accepted by both players and coach. By contrast, in a controlling environment, athletes are less involved in the decision making process and receive less explanatory rationale of their coach [22,24]. As a result, the development of athletes’ knowledge is not guaranteed and the chance of misunderstood decisions increases.

Cross-sectional studies already demonstrated the positive link between need support and (procedural) justice in both business [30] and sport settings [6]. Apart from the fact that this is the first longitudinal study that examined the relationship between need support and perceived justice, the link between a controlling coaching style and justice has not yet been examined.

### Game-specific predictor of perceived justice: Decision justifications

Besides the more general need supportive coaching style, the present study also focused on a game-specific and thus more fluctuating coach behavior, namely the provision of decision justifications. Decision justifications have been defined as “explanations in which the actor admits personal responsibility for the action, but minimizes or denies its severity as perceived by the audience” [28] (p. 474). The actor can do this by appealing to superordinate values and goals in order to reframe the event or its consequences. For example, the coach can explain that the limited number of playing minutes of a certain player was the consequence of his tactical decision (i.e., admitting personal responsibility) in order to maximize the chances for the team to win the game (i.e., reframing the consequences of the decision). Decision justifications have been found to be positively related to perceived justice in the business setting. Two reasons were mentioned to explain the positive effect of credible explanations on perceived justice: they convey respect from authorities, and they provide information about how and why decisions are made [28]. Regarding attribution research, people who encounter unfavorable, negative or unexpected decisions, are more motivated to search for causal information and consequently question the why of decisions [31]. In line with this, several studies showed that the effect of adequate and sincere decision justifications on perceived justice is even bigger when employees consider an event or decision as being unexpected, controversial or negative [28,32,33].

It is important to consider that coaches provide such justifications as a form of rationale *after* the decision has been made [34]. The fact that justifications are post hoc explanations distinguishes them from most need supportive behaviors. Need supportive coaches try to involve athletes in the decision making process; take their opinions and perspectives into account; and structure expectations or instructions *before* decisions are made [16,19].

### Game-specific predictors of perceived justice: Athletes’ status and game result

Finally, we aimed to study two game-specific predictors of athletes’ perceived justice that were not directly related to the coach behavior. In particular, we examined whether (a) athletes’ status during the game, and (b) the result of the game predicted players’ perceived justice. Aside from framing the outcome of these predictors as being a personal (starter vs. substitute) or team aspect (win vs. loss), players may perceive the outcome itself as either being favorable (starter/win) or unfavorable (substitute/loss). As each team consists of starters and substitutes and will have varying results, both are inevitable and unchangeable factors with which the coach has to deal in the specific context of sport teams.

Until now, no studies have evaluated the impact of the status of the athlete on his/her perception of coaches’ justice. In this light, previous research in other domains found evidence for the so-called self-serving bias [35]. This bias refers to the general tendency for people to perceive as fairest those outcomes that best serve their interests. For example, Greenberg [36] demonstrated that nonsmokers perceived a smoking ban in their company as more fair than smokers did. In addition, it is generally acknowledged that procedures are judged as more fair when they result in favorable outcomes [35]. In addition to the self-serving bias, Holtz and Harold [7] suggested that the affective events theory [37] could be incorporated to explain employees’ justice judgements. Affect influences the type of information individuals attend to, interpret and recall. Forgas and George [38] stated that affect selectively activates memory structures to which it is connected, facilitates the recall of information encountered in a matching mood state and strengthens the use of mood-congruent associations in the interpretation of ambiguous social information. They conclude that these processes lead to a mood-consistent bias in organizational judgments. For example, a person in a good mood could interpret a smile as friendly and cooperative, while a person in a bad mood sees that same smile as superior and condescending [38]. Building on those findings, we assume that the degree to which the decisions were perceived as beneficial to themselves (i.e., being a starting player) would have a positive impact on players’ affect, and in turn would be positively related to athletes’ justice perceptions. In other words, the coach might be perceived as more fair by the starting players compared to the substitutes because he/she decided in the starting player’s favor.

Second, we examined to what extent the result of the game predicted the fluctuations of athletes’ perceived justice. Whisenant and Jordan [39] indicated that players’ justice perceptions of teams with winning records were higher compared with the perception of those with losing records. Elaborating on Whisenant and Jordan’s findings [39], we studied the weekly fluctuations of this relationship. Assuming that losing is a negative experience and based on the previously described attribution research [31] and the affective events theory [37], we hypothesized that players report lower levels of perceived justice of their coach after losing a game compared to after winning a game.

### Current study

Considering the positive relationship between athletes’ perceived justice of the coach and team functioning, we were interested in the game-to-game fluctuations of justice and its predictors. This within-player variability in perceived justice of the coach has not yet been studied. However, considering that within person variance accounted for approximately one third of the variance of supervisory justice in the business setting [7], we expected significant variability in athletes’ perception of justice across a period of six weeks. In the current study, we checked whether fluctuations of justice were linked to coach and game-related predictors.

A need supportive coaching style is the only predictor that already was linked to team athletes’ perception of justice [6]. In the current study, we aimed to confirm our previous cross-sectional findings in a longitudinal design. Furthermore, by adding the much less studied controlling coaching style, we aimed to contribute to the SDT literature. In line with previous cross-sectional results and the links between SDT and the above mentioned strategies to increase perceptions of justice, we hypothesized that the general coaching style would be substantially related to athletes’ perceived justice of their coach.

*Hypothesis 1a*: *A perceived need supportive coaching style is positively related to athletes’ game-specific perception of justice*.

*Hypothesis 1b*: *A perceived controlling coaching style is negatively related to athletes’ game-specific perception of justice*.

Furthermore, elaborating on results in the business setting [32, 33], we expected that providing justifications after the game was positively linked to athletes’ game-specific perceived justice. Moreover, we decided to look at the interaction effects between need support and control on the one hand and coaches’ decision justifications after the game on the other hand. In a need supportive environment, coaches provide exploratory rationales and players get the opportunity to voice their concerns [16,18,19]. As a result players and coaches will share a clear framework resulting in more predictable decisions. In contrast, when the coach uses a controlling coaching style athletes’ voice is restricted and rationale is not always provided [16,24]. Therefore, the provision of justifications could be a moment of clarifying communication and could compensate for the limited input and explanations before the event. Consequently, when coaches use a controlling style, decision justifications could have a stronger impact on the weekly fluctuations of athletes’ perceived justice.

*Hypothesis 2a*: *The weekly fluctuations of perceived justice are less strongly related to decision justifications when players perceive high levels of need support compared with when they perceive low levels of need support*.

*Hypothesis 2b*: *The weekly fluctuations of perceived justice are more strongly related to decision justifications when players perceive high levels of psychological control compared with when they perceive low levels of psychological control*.

The final aim of the study was to investigate the link between athletes’ perceived justice of their coach and two game-specific circumstances: the status of the players, and the game result. Based on the self-serving bias [35], attributional research [31] and the affective events theory [37], we expected that players’ perception of justice is positively related to being a starting player and winning games.

*Hypothesis 3a*: *Players reported higher levels of perceived justice in the weeks they are part of the starting team compared to the weeks in which they were substitutes*.

*Hypothesis 3b*: *Players reported higher levels of perceived justice in the weeks their team win the game compared to the weeks in which their team lose the game*.

## Method

### Participants

Participants were 57 female volleyball and 39 male handball players from the highest two divisions in the Belgian league. The female volleyball players were recruited from nine teams, seven of the eight Flemish first division teams and two teams in the second division. The male handball players were recruited from seven teams, all five Flemish first division teams and two second division teams. Spread over the 16 teams, 96 of the 204 athletes completed the questionnaires (i.e., 47.1%). However, the response rate for the volleyball players was significantly higher (i.e., 57.6%) than that of the handball players (i.e., 37.1%). This can be attributed to two reasons. First, relatively more foreign athletes played in the male handball teams. Because the questionnaire was drawn up in Dutch, the non-Dutch-speaking players did not participate in the study, this could have led to a lower response rate. Second, the coach of the national female volleyball team was one of the researchers, which might have stimulated them to participate. We would emphasize that we have followed sound scientific procedures to assure that our research was performed according to the strictest possible ethical standards. First, at the start of the study complete anonymity was assured. Players chose a codename to fill in the questionnaire and none of the researchers could link names to the data. Second, none of the players who filled in the questionnaires was part of the national team or trained under supervision of the national team coach. Finally, players only filled in questions about their own coach, none of the questions handled about the coach of the national team. Participants were clearly informed about these procedures to avoid biased results.

The players were on average 23.15 (SD = 4.47) years old. They had been playing volleyball or handball on average for 13.58 years (SD = 4.91), worked with the same coach for 2.48 years (SD = 3.16) and trained 2.92 times (SD = 0.91) per week. Those demographical variables did not differ between the female volleyball and the male handball players.

### Measures

Data were collected with two different questionnaires. The general questionnaire was used the first week of the study and gathered information about the players’ demographics and the general coaching style. The game-specific questionnaire had to be completed after each game during six consecutive weeks and gathered information about athletes’ perceived justice of the coach, decision justifications, players’ status, and the game result. Items were answered on a 5-point Likert scale ranging from 1 (strongly disagree) to 5 (strongly agree).

### General questionnaire: Overall coaching style

#### Need support of the coach (9 items)

In line with Deci and Ryan [12] we considered need support of the coach as a concept composed of three subscales: (a) autonomy-support, (b) competence-support and (c) relatedness-support. To assess the concept of need support we selected nine items from two different questionnaires which were already translated into Dutch and used in the sport setting by De Backer et al. [4,6]. An example of such an autonomy support item was: “My coach let me partially decide on my own training program”. An example of a competence support item is: “I have the impression that my coach believes in my abilities”. Finally, and example of a relatedness support item is: “My coach shows that he/she cares about me as a person”.

#### Control of the coach (3 items)

To assess athletes’ experience of coaches’ controlling coaching style we included items that tapped coaches’ coercive behavior. Based on the study of Pelletier and colleagues [40] three items were back-translated into Dutch and adapted to fit the elite team sport context. An example item is: “My coach makes me feel guilty when I do not meet his/her expectations”.

### Game-specific questionnaire

#### Decision justification (4 items)

The 4-item scale was based on research about the impact of decision justifications within business settings [13]. Greenberg’s items questioned the explanation, communication, and the clearness of the reasons of pay cuts. We transformed them to fit the team sport setting and queried the clarity and the communication of the tactical decisions and the player’s selection (e.g., “My coach explained his/her tactical decisions”).

#### Perceived justice (12 items)

This section was based on research of Colquitt [41] and adapted to the sport context by De Backer et al. [4]. Justice was considered as a concept composed by four subcategories of three items: personal distributive justice, group distributive justice, personal procedural justice, and group procedural justice. The personal distributive justice category questioned the perceived justice of the individual playing time during the game (e.g., “My coach rewarded me with a fair amount of playing time taking into account my contribution to the team”). The group distributive justice category informed about the outcomes for the group as a whole (e.g., “My coach based his selection of the starting team on the talent and competence of the players”). The personal procedural justice category referred to the perceived justice of the procedures used by the coach for the players’ individual treatment (e.g., “The evaluation of my performance during the game and/or training was supported by reliable information”). Finally, the group procedural justice category questioned the procedures the coach used towards the group as a whole (e.g., My coach consequently substituted players when they were underperforming).

Despite the fact that people can distinguish between these sources of justice, Greenberg [42] (p. 211) suggested that individuals form justice perceptions based on a ‘‘holistic judgment in which they respond to whatever information is both available and salient”. Moreover, researchers have shown that individual’s justice perceptions may not be accurately evaluated when there is a focus on the various dimensions of justice [43]. Finally, De Backer et al. [4] indicated that the different justice subcomponents form a latent variable of overall justice, which was linked to identification and cohesion in a team sport context. Therefore, we combined the four subcomponents and shifted towards an overall justice judgment.

#### Status of the athlete (1 item)

With a single item, we questioned whether the player was a member of the starting team (i.e., “During last game I was a starting player / a substitute”).

#### Result of the game (1 item)

With a single item, we questioned the result of the game (i.e., “Last game our team won/lost.”).

### Procedure

The head coaches of the teams were contacted and informed about the aim of the research. Only when the coach gave his/her permission, a research assistant informed and invited the players to participate by means of a brief verbal presentation during or after a training session. The players who agreed to participate, were sent an e-mail in which they were informed about the timing and the objectives of the web-based questionnaires. Furthermore, they were guaranteed full anonymity. We collected data during six consecutive mid-season weeks. The first assessment took place after the first game of these six weeks with the aim of assessing general measures (i.e., demographical information and the coaching style). The other assessments took place after each game during six consecutive weeks and collected game-specific measures (i.e., within-athletes measures including perceived justice, decision justifications, the game specific status of the player, and the game result).

An email, with the link to the questionnaire, was sent on a weekly base on Sunday. Moreover, the players who had not completed the questionnaire on Wednesday, received a reminder email. Finally, when the questionnaire was not filled out by Friday, the players were reminded a last time by phone.

As part of a bigger PhD project [44], the current study was approved by The Leuven International Doctoral School Biomedical Sciences. More specific, by the Doctoral Committee of Kinesiology, Rehabilitation Sciences & Physiotherapy. The research conducted was in line with the ethical principles of the American Psychological Association (APA). No rewards were given for participation, informed consent was obtained from all participants, and confidentiality was assured.

### Data analysis

First, we performed Confirmatory Factor Analysis (CFA) to test whether the back-translated and slightly adapted measurement instruments fitted the already validated structure of the original measurement models. The indices we used to evaluate overall model fit were: comparative fit index (CFI), Tucker–Lewis Index (TLI), and root mean square error of approximation (RMSEA A CFI and TLI value higher than .90 [45], and a value lower than .08 for the RMSEA [46] indicate an acceptable fit of the model. Consequently, internal consistencies of the different scales were calculated. Finally, Hierarchical Linear Modeling (HLM) with R was conducted to examine the fluctuations of perceived justice and the effect of the above-mentioned determinants on athletes’ perceived justice of the coach in a longitudinal perspective.

#### Plan of analysis

Our game-to-game measures (i.e., level 1 or within-athlete variance) were nested into individuals (i.e., level 2 or between-athlete variance). Therefore, it was crucial that our statistical model explicitly recognized this hierarchical structure and that variation at within and between athlete level was allowed for in the analyses. Multilevel modelling (using R) provides an efficient way of doing this. Furthermore, these multilevel models allowed us to model cross-level interactions (Hypothesis 2a and 2b). An overview of the models can be found in Table 1. First, we compared a model with only level 1 variance to a model with both level 1 and level 2 variance and checked whether the addition of the second level significantly improved our model. We computed the Intraclass Correlation Coefficient (ICC) to examine the degree of variance underlying the within and the between-athlete level.

**Table 1:** The multilevel models aiming to address the different hypotheses of the study.

What is the game-to-game variance in players’ perceived justice of the coach? – Intrapersonal level: y_ij_ = β_0j_ + r_ij_ – Interpersonal level: β0j = γ00 + u0j
H1a and 1b: Does the general coaching style predict players’ perceived justice of the coach? – Intrapersonal level: y_ij_ = β_0j_ + r_ij_ – Interpersonal level: β_0j_ = γ_00_ + γ_01 [need support]_ + γ_02 [control]_ + u_0j_
H2a and 2b: Do the weekly decision justifications predict players’ perceived justice of the coach? And does the interaction effect of decision justifications and the coaching style have an additional effect on players’ perceived justice of the coach? – Intrapersonal level: y_ij_ = β_0j_ + β_1j[justifications]_ + r_ij_ – Interpersonal level: β_0j_ = γ_00_ + γ_01 [need support]_ + γ_02 [control]_ + u_0j_ β_1j_ = γ_10_ + γ_11 [need support]_ + γ_12 [control]_
H3a and 3b: Do game-specific circumstances (i.e., athletes’ status & game result) have an additional effect on players’ perceived justice of the coach? – Intrapersonal level: y_ij_ = β_0j_ + β_1j[justifications]_ + β_2j[athletes’ status]_ + β_3j[game result]_ + r_ij_ – Interpersonal level: β_0j_ = γ_00_ + γ_01 [need support]_ + γ_02 [control]_ + u_0j_ β_1j_ = γ_10_ + γ_11 [need support]_ + γ_12 [control]_ β_2j_ = γ_20_ β_3j_ = γ_30_

In order to check Hypothesis 1a and 1b, we tested whether the coaching style (i.e., level 2) predicted athletes’ game-to-game perceived justice of the coach. We entered both the need supportive and the controlling coaching style (grand mean centered) as predictors at level 2 (i.e., between-athletes).

Regarding Hypothesis 2a and 2b, the weekly decision justifications of the coach (group mean centered), were added to our previous model as a predictor at level 1 (within-athletes). Furthermore, the model was completed by adding the two cross-level interaction effects between both coaching styles one the hand and decision justifications on the other hand.

To examine Hypothesis 3a and 3b we extended the model of the previous step. Two game-specific circumstances were entered as predictors at level 1, namely the status of the player and the result of the game.

## Results

### Descriptive statistics, correlations, and scale reliabilities

See Table 2 for descriptive statistics and bivariate correlations. In order to correlate level 1 and level 2 variables, we calculated the mean score of the level 1 variables based on the six weekly scores. Scale reliabilities of the general and the first weekly questionnaire are provided within parentheses on the diagonal. Means and standard deviations of perceived justice and decision justification across the six matches can be found in the appendix.

**Table 2:** Means, standard deviations correlations and Cronbach’s alphas for all variables.

	Variable	M	SD	1	2	3	4
1.	Need supportive coaching style	3.20	.74	*(*.*84)*			
2.	Controlling coaching style	2.36	.86	-.34**	*(*.*64)*		
3.	Game-specific decision justifications	3.60	.75	.41**	-.22*	*(*.*89)*	
4.	Game-specific perceived justice	3.20	.52	.44**	-.24*	.79**	*(*.*75)*

5-point Likert-type scale; aggregated score across the six games for game-specific decision justifications and perceived justice (i.e., mean score based on the scores for the six games).

* p < .05

** p < .01

### CFA perceived justice, coaching style, and decision justifications

It is critical that the measurement of the different factors is psychometrically sound [47]. Therefore, we tested the factorial structure of each scale via CFA. The CFA on the proposed four-factor model for athletes’ perceived justice of the coach provided acceptable fit to the data (χ^2^ = 240.88, *df* = 49, *p* = .00; CFI = .94; TLI = .92; RMSEA = .08). All items loaded significantly on their respective subscale (ranging from .20 to .93). Furthermore, the four subscales loaded significantly on the overall justice factor (ranging from .76 to .99). We computed the mean of the 12 items to acquire an overall perceived justice rate. Furthermore, CFA supported the proposed four-factor model (i.e., autonomy, competence, and relatedness support, and control) for the overall coaching style (χ^2^ = 59.58, *df* = 40, *p* = .02; CFI = .95; TLI = .93; RMSEA = .07). All factor loadings (.38 - .93) were statistically significant except one. The non-significance of this autonomy item could possibly be explained by the negative formulation of the item (i.e., *“My coach does not care about my point of view on training and games”*). We deleted this reversed item. Furthermore, the autonomy, competence and relatedness factor were significantly related to the second order need supportive factor (.73–1), which was in turn negatively correlated with the control factor (*r* = -.46). We computed the mean of the eight items to acquire a need supportive coaching style measure and the three items to acquire a controlling style. Finally, CFA supported the one-factor model for decision justifications (χ^2^ = 2.11, *df* = 1, *p* = .15; CFI = 1; TLI = 1; RMSEA = .04). All factor loadings were statistically significant and ranged from .77 to .87. We computed the mean of the four items to obtain a decisions justifications rate.

### Main analyses

#### Variability of athletes’ game-specific perception of justice

We examined the degree of variability in players’ game-specific perception of justice of their coach. To start, we checked whether the addition of the level 2 significantly improved our model. Therefore, we compared a model with only within-athlete variance to a model with both within and between-athlete variance. The likelihood ratio test indicated that the addition of the between-athlete level significantly improved the model (Δχ^2^ (2) = 271.11, *p* < .001). This likelihood ratio expresses how many times more likely the data are under one model than the other. Inspection of the intercept of the two-level model showed that the average reported perceived justice of the coach was 3.19. Furthermore, the ICC for athletes’ perception of justice of the coach was ρ = .51, suggesting that the total variability consists of almost as much within-athlete variability (i.e., 49% variability within one player during the six weeks) as between-athlete variability (i.e., 51% variability between the different players). In other words, athletes’ perception of justice varies approximately as much between different games of the same player as between different players.

#### Predicting perceived justice from the coaching style (Hypothesis 1a, 1b)

We calculated whether a need supportive or a controlling coaching style (i.e., level 2) predicted athletes’ perception of game-specific justice of the coach. The model had an intercept of 3.19 and the calculations showed that a need supportive style was a significant positive predictor γ01 [need support] = .28, SE = .07, *p* < .001, while a controlling style was not significantly related to perceived justice γ01 [control] = -.06, SE = 06, *p* = .287. This model accounted for 21% of the variance in athletes’ initial justice perceptions (pseudo *R*^2^ = .21). Moreover, this model fitted significantly better than the two-level model used to calculate the ICC (i.e., Δχ^2^ (2) = 19.9, *p* < .001). We can conclude that a need supportive coaching style increased athletes’ justice, while a controlling coaching style not significantly decreased athletes’ perceived justice.

#### Predicting perceived justice from game-specific coach behavior (Hypothesis 2a, 2b)

In the next step, we added the game-specific decision justifications on level 1 to the multilevel model of the previous step and tested whether it predicted weekly fluctuations of athletes’ perceived justice. Moreover, in order to get a complete view of the dynamical influence of the coaching style and decision justifications we added the interaction effects between both coaching styles and coaches’ justifications to the above described model. Results showed that the need supportive style remained a significant predictor and the controlling style remained a non-significant predictor of justice when decision justifications were added, γ01 [need support] = .27, SE = .07, *p* = .001; γ02 [control] = -.07, SE = .06, *p* = .260. Furthermore, decision justifications positively predicted athletes’ perceived justice: γ10 [justifications] = .34, SE = . 02, *p* < .001. The interaction effect between a need supportive coaching style and coaches’ justifications γ11[need support x justifications] = -.05, SE = .03, *p* = .126 was not significant. However, the interaction effect between a controlling coaching style and decision justifications was significant γ12 [control x justifications] = .06, SE = .03, *p* = .036. The addition of decision justifications and the interaction effects explained 29% of the within-athletes’ variance of overall justice perceptions (pseudo *R^2^* = .29).Finally, adding the game-specific decision justifications improved our previous model significantly (i.e., Δχ^2^ (3) = 199.2, *p* < .001).

#### Predicting perceived justice from game circumstances (Hypothesis 3a, 3b)

We extended the multilevel model used in the previous step. More specifically, we added two game-specific circumstances on level 1: athletes’ status (i.e., 0 = starting player; 1 = substitute), and the game result (i.e., 0 = victory; 1 = loss). Our final model had an intercept of 3.94 and showed that need support and decision justifications remained significant predictors of justice when the game-specific predictors were added, γ01 [need support] = .28, SE = .07, *p* < .001; γ10 [justifications] = .29, SE = . 02, *p* < .001. The link between a psychological controlling coaching style and perceived justice remained non-significant γ02 [control] = -.06, SE = .06, *p* = .286. Furthermore, the model showed that being a substitute and losing a game negatively predicted perceived justice: β2j[athletes’ status] = -.38, SE = . 05, *p* < .001, and β3j[game result] = -.16, SE = . 04, *p* < .001.

**Fig 1:**
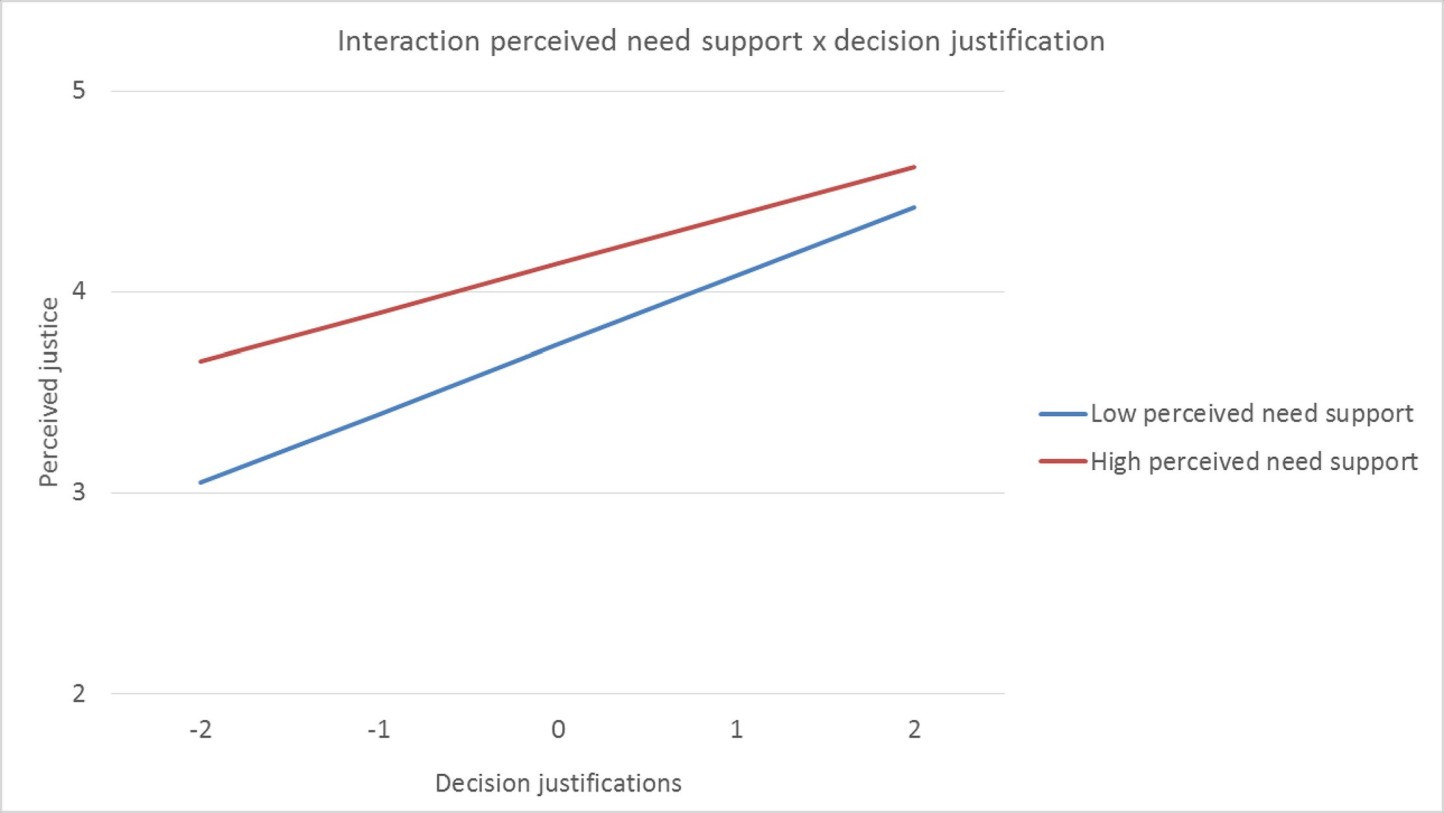
Plot of the cross-level interaction effect between need support and decision justifications. Perceived justice on the Y-axis, decision justifications on the X-axis (group-mean centered).

**Fig 2:**
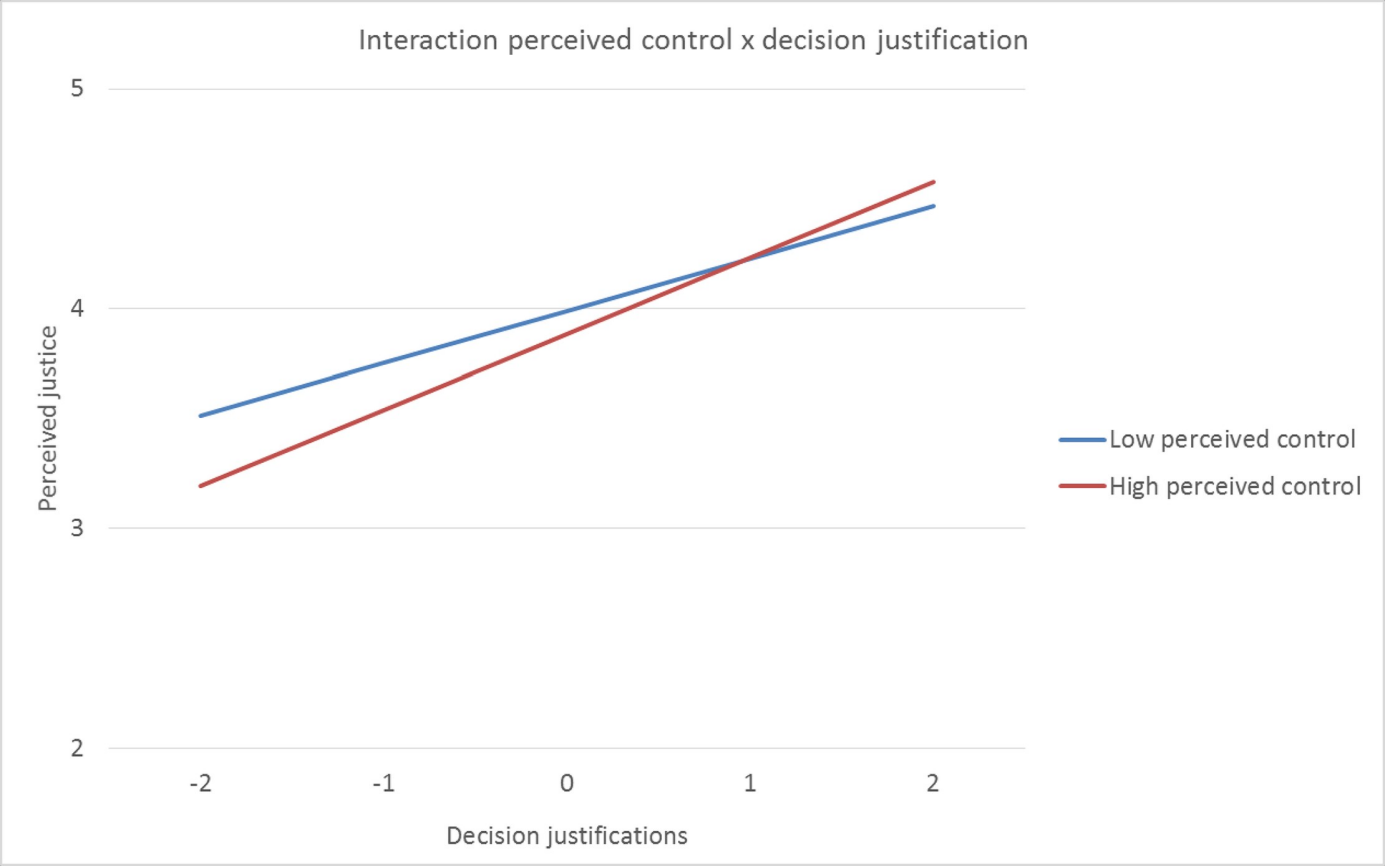
Plot of the cross-level interaction effect between control and decision justifications. Perceived justice on the Y-axis, decision justifications on the X-axis (group-mean centered).

Interestingly, the interaction effect between a need supportive coaching style and justifications became significant when game specific circumstances were added to the model γ11 [need support x justifications] = .07 SE = .03, *p* = .036 The interaction effect between a controlling coaching style and justifications remained significant when game specific circumstances were added to the model γ12 [control x justifications] = .07 SE = .03, *p* = .023. Simple slope analyses revealed that when players’ perceived a higher level of need support, decision justifications did less strongly predict perceived justice (β = 0.24, *SE* = 0.04, *p* < .001), than when players’ perceived a lower level of need support (β = 0.34, *SE* = 0.03, *p* < .001). Contrary, when players’ perceived control was higher, decision justifications did more strongly predict perceived justice (β = 0.35, *SE* = 0.03, *p* < .001), than when players’ perceived control was lower (β = 0.24, *SE* = 0.04, *p* < .001). A graphical representation of these interaction effects can be found in Fig 1 and Fig 2. This final model additionally explained 11% of the within-athletes’ variance of overall justice perceptions (pseudo *R^2^* = .11). Finally, adding the game-specific circumstances improved the model significantly (i.e., Δχ^2^ (2) = 73.7, *p* < .001). The results of this final model can be found in Table 3.

**Table 3:** Prediction of perceived justice as a function of perceptions of a need supportive/controlling coaching style, and weekly decision justifications, athletes’ status, and game result.

Fixed effects		Perceived justice
Intercepts (between athletes)		
Intercept,	γ_00_	3.93 (0.10)
Need supportive coaching style,	γ_01_	0.28* (0.07)
Psychological controlling coaching style,	γ_02_	-0.06 (0.06)
Decision justification Slopes (within athletes)		
Direct effect,	γ_10_	0.29* (0.02)
Cross-level interaction need support,	γ_11_	-0.07* (0.03)
Cross-level interaction control	γ_12_	0.07* (0.03)
Starter vs. Non-starter slopes (within-athletes)		
Direct effect,	γ_20_	-0.38* (0.05)
Win vs. Loss slopes (within-athletes)		
Direct effect,	γ_30_	-0.16* (0.04)
Random effects		Variance components
Intercept,	u_oj_	0.19 (0.44)
Within-athlete,	r_ij_	0.14 (0.38)

* *p* < .05

## Discussion

Recent research in the team sport context has shown that need support of the coach is positively related to athletes’ perceived justice of the coach, which in turn plays an important role in optimizing the team’s functioning [5]. Despite these promising results, sport scientists have neglected the fact that players’ perception of justice probably fluctuates from week to week. Therefore, the current study approached athletes’ perception of justice from a longitudinal point of view. This allows for a more dynamic consideration of athletes’ perceived justice and provides the opportunity to examine its link with game-specific predictors such as decision justifications of the coach, athletes’ status, and the game result.

First, our results indicated that athletes’ perception of justice is a dynamic concept which varies approximately as much between different games of the same player as between different players. This result contradicts the uncertainty management theory, which assumes that overall justice perceptions are resistant and that change will occur only in the face of relatively dramatic events, such as changes in leadership or organizational restructuring [9]. The strong fluctuations in justice perceptions could be the result of the specific environment of elite sport teams, characterized by weekly and publically evaluations and continuously changing game-circumstances. Taking into account the current longitudinal findings and the previous results of Holtz and Harold [7], we suggest to no longer ignore the longitudinal fluctuations of perceived justice.

Second, by demonstrating a positive relationship between a need supportive coaching style and athletes’ game-specific perceived justice of the coach at the between-athletes level (i.e., Hypothesis 1a), our longitudinal study confirmed the results of the cross-sectional study of De Backer et al. [6]. These results seem to be a logical consequence of the fact that need supportive coaches (a) allow their athletes to express their views, (b) offer relevant information to their athletes, and (c) care for their athletes by supporting their progression and treating them with dignity and respect [16, 18, 19]. In addition, previous research indicated that in a need supportive environment athletes develop higher levels of tactical knowledge [29]. As a result, the probability of misinterpreting decisions decreases. This could partially explain why need support enhances athletes’ perception of justice. For example, when athletes understand the tactical motives behind the selection of the starting team, they are more willing to accept this selection and perceive it as fair. Furthermore, we aimed to increase the knowledge about the under examined need-thwarting side of the SDT-continuum. In contrast to Hypothesis 1b, our findings showed a non-significant negative link between a controlling coaching style and athletes’ perception of justice of their coach. The impact of a controlling coaching style will be discussed in depth in the following paragraphs.

Third, in line with cross-sectional research in the business setting [33], our results showed that decision justifications positively predict athletes’ perceived justice of the coach. Due to the longitudinal perspective we can conclude that the weekly provision of decision justifications is substantially related to the within-athletes’ fluctuations of perceived justice.

Moreover, in line with Hypothesis 2a and 2b, the final model showed significant interaction effects between a need supportive and a controlling coaching style on the one hand and justifications on the other hand. We can conclude that the impact of perceived decision justifications on the weekly fluctuations of athletes’ perceived justice was lower when the coach used a strong need supportive style and higher when he/she used a strong controlling style. This relation is in line with previous attribution research [31] and can probably be explained by the statement that justifications have a bigger effect when decisions are unexpected, negative or unfavorable [28, 32]. As already described above, in a need supportive environment coaches do actively involve their athletes in the training process, which increases athletes’ insight in the decision framework and gets the coach and the players on the same page [12, 29]. As a result, the chance that unexpected events occur decrease. On the contrary, in a controlling environment the communication between athletes and coaches is often unilateral and less focused on the explanatory rationale behind decisions [16, 24]. Consequently, players and coaches do not automatically share the same decision framework, which increases the risk of misunderstood and unexpected decisions. Mouratidis et al. [22] already suggested that a controlling coach risks to be perceived as unfair. As a result, in a strong controlling climate players are more sensitive to decision justifications.

Fourth, confirming Hypothesis 3a and 3b, the status of the player and the result of the game showed additional links with athletes’ game-specific perceived justice of the coach. In line with previous research on the self-serving bias of perceived justice [35, 36] and the affective event theory [37], we found that players who were in the starting team or won a game reported higher levels of perceived justice of their coach after the game. It seems that the degree to which the decisions were perceived as favorable to themselves was positively related to athletes’ justice perceptions. Despite the fact that we did not measure athletes’ affect, these last findings are in line with previous results of studies about the affective events theory [37]. The negative affective state caused by not being in the starting team or by losing the game could possibly explain why these specific game circumstances were negatively linked to players’ perceived justice of the coach.

From a practical point of view, our findings showed that coaches (i.e., general coaching style and the weekly provision of decision justifications) are key predictors of athletes’ perceived justice. In particular, when athletes perceived their coaches as need supportive they also perceived them as more fair. In addition, our results indicate that especially strong controlling coaches benefitted considerably from providing decision justifications. Besides the coach, also the weekly fluctuating game circumstances such as losing a game or being a substitute predicted athletes’ perceived justice of the coach. However, even in the ‘worst-case-scenario’ in which the player is a substitute and loses the game, coaches can buffer this negative effect of the game circumstances on athletes’ perceived justice by obtaining a need supportive general coaching style and providing decision justifications.

Previous research have been indicating substantially links between athletes’ perceived justice and higher levels of team commitment, team identification, and team cohesion and lower levels of social loafing [3, 5]. Therefore, we concluded that coaches should attempt to keep athletes’ justice perception high and limit its fluctuations across the season in order to optimize the functioning of elite sport teams.

A first practical recommendation that we would provide to coaches is to create a need supportive environment. Mageau and Vallerand [18] (p. 898) stated that “to satisfy all three psychological needs, autonomy-supportive behaviors need to be conveyed within a specific structure and accompanied by high levels of involvement”. Thus, it is not enough to offer athletes’ some choice and freedom. Coaches also need to provide a clear structure and set limits within which athletes get some responsibility about their own learning process. For example, coaches can proactively clarify the selection of the starting team based upon transparent tactical principles. In addition, they can objectively and with respect for their athletes’ disappointment discuss lost games and turn the loss into new challenging and shared goals. Our results have shown that such a need supportive coaching style partly buffered the negative effect of losing a game or being a substitute on athletes’ perceived justice.

However, personal characteristics of the coach and specific situations in elite team sport settings, could ask for a less need supportive approach of the coach. Sometimes, he/she may be forced to behave in a more controlling way. For example, time pressure can arise when games have to be played in quick succession, leaving no time for consultation or discussion between the coach and the players. In addition, sometimes the coach has to force players to put the benefit of the team above their own personal benefits. Our results have shown that a controlling coaching style is not overly negative for athletes’ perceived justice, but coaches have to realize the possible pitfalls of this coaching style. For example, when using a controlling style, coaches can less rely on a shared and commonly agreed framework. As a result, the provision of clear decision justifications is even more crucial to ensure that athletes and coaches stay on the same page.

As with any research, the current study had some limitations. First, we focused on distributive and procedural justice and did not measure the interactional justice subcomponent (i.e., interpersonal and informational justice). Future research should examine whether our results could be replicated when this form of justice is added to the overall justice measurement. However, a need supportive coaching style and decision justifications are characterized by treating players with respect and providing information. As a result, we expect that the relationships would be even stronger when we add interactional justice.

Second, in order to measure the general coaching style (need supportive or controlling) and the game-specific decision justifications, we had to rely on measures that were not yet specifically validated to the context of sport. Therefore, we have performed CFA’s that provided preliminary psychometrical support for the different scales. However, the rather low Cronbach’s alpha of the controlling coaching style measure (3 items) remains a point of concern.

Third, despite the longitudinal perspective, our study was only a first step in the understanding of the dynamical process of athletes’ perceived justice of their coach. As mentioned earlier, unexpected events are perceived as less fair compared to expected events. It is possible that the extent to which the status of the player or the result of the game is unexpected would have a bigger impact on athletes’ perceived justice than the direct status or result itself. It would be interesting to include players’ expectations in future studies.

Finally, the current study examined the mean effect of losing a game or being a substitute on players’ perceived justice of the coach, but it did not check whether the strength of these effects varied during the six weeks. An in-depth analysis could further inform us whether the strength of those predictors is affected by the recent history of the player (e.g., regular substitute, or only in this particular game) and the team (e.g., first loss in a long time, or a series of losses).

### Perspective

Being perceived as fair is crucial for coaches in order to obtain optimal team functioning [3,5]. Previous studies already related need support from the coach to perceived justice in a cross-sectional design [4]. In order to capture the dynamical nature of sport teams, the current study was the first to use a longitudinal approach to examine perceived justice in team sport settings and its relation to coaching style and game circumstances. Our results showed that athletes’ perception of justice of their coach shifts considerably from game-to-game. Based on our findings we conclude that the coach is a crucial predictor of the degree of the weekly fluctuations of athletes’ perceived justice. Moreover, the coach seems to be able to overcome the negative effects of being a substitute or losing a game. In order to optimize athletes’ justice perception and decrease the fluctuations of justice, we would recommend coaches of elite sport teams to be need supportive when the situation allows it, and to provide justifications when the situation requires it.

## Supporting information

S1 Table Means, and standard deviations for game-specific decision justifications and perceived justice of the six games. (DOCX)

S1 File General and weekly questionnaire. All items used in our study (English and Dutch).
(DOCX)

S2 File Level 1 variables. SPSS file off all the level 1 variables. (SAV)

S3 File Level 2 variables. SPSS file off all the level 2 variables.
(SAV)
