# Supplementary material for: Do coaching style and game circumstances predict athletes' perceived justice of their coach? A longitudinal study in elite handball and volleyball teams
Source: PLoS One. 2018 Oct 15;13(10):e0205559. doi: 10.1371/journal.pone.0205559 (PMC6188870; doi:10.1371/journal.pone.0205559)
Supplement: S1 Table — (DOCX) [file pone.0205559.s001.docx]

**S1 Table. Means, and standard deviations for game-specific decision justifications and perceived justice of the six games.**

|  | Game | Game-specific decision justifications | | Game-specific perceived justice | |
| --- | --- | --- | --- | --- | --- |
|  |  | M | SD | M | SD |
| 1. | Game 1 | 3.68 | 1.03 | 3.05 | 0.58 |
| 2. | Game 2 | 3.69 | 0.97 | 3.27 | 0.63 |
| 3. | Game 3 | 3.57 | 0.96 | 3.19 | 0.72 |
| 4. | Game 4 | 3.67 | 1.00 | 3.31 | 0.68 |
| 5. | Game 5 | 3.59 | 0.91 | 3.27 | 0.68 |
| 6. | Game 6 | 3.72 | 0.89 | 3.30 | 0.67 |
