# Supplementary material for: Do coaching style and game circumstances predict athletes' perceived justice of their coach? A longitudinal study in elite handball and volleyball teams
Source: PLoS One. 2018 Oct 15;13(10):e0205559. doi: 10.1371/journal.pone.0205559 (PMC6188870; doi:10.1371/journal.pone.0205559)
Supplement: S1 File — All items used in our study (English and Dutch). (DOCX) [file pone.0205559.s002.docx]

**General Questionnaire**

**Need supportive coaching style**

| *My coach let me partially decide on my own training program.*  Mijn coach laat mij gedeeltelijk mee beslissen over mijn eigen trainingsprogramma. |
| --- |
| *My coach does not care about my point of view on training and games.*  Mijn coach is niet erg gevoelig voor wat ik belangrijk vind op training of wedstrijd. |
| *My coach is usually willing to take my point of view into consideration.*  Mijn coach is meestal bereid om de zaken vanuit mijn standpunt te bekijken. |
| *My coach provides positive feedback to me.*  Mijn coach geeft mij positieve feedback. |
| *I have the impression that my coach believes in my abilities.*  Ik heb de indruk dat mijn coach gelooft in mijn capaciteiten. |
| *My coach confirms confidence in my volleyball abilities to successfully fulfill an exercise.*  Mijn coach bevestigt mijn vertrouwen in mijn volleybalcapaciteiten om een oefening tot een goed einde te brengen. |
| *My coach shows that he/she cares about me as a person.*  Mijn coach toont interesse in mijn persoonlijke noden en behoeften als mens. |
| *I can talk with my coach about non-volleyball issues that are important to me.*  Ik kan bij mijn coach terecht om over andere dingen te praten dan volleybal die voor mij belangrijk zijn. |
| *My coach is willing to support me when I have problems that are not sport-related.*  Ik kan ook met mijn problemen buiten het volleybal bij mijn coach terecht. |

**Psychological controlling coaching style**

| *My coach clearly shows that he/she personally feels hurt if I do not meet his/her expectations.*  Mijn coach laten duidelijk merken dat hij zich persoonlijk gekwetst voelt als ik niet voldoe aan zijn verwachtingen. |
| --- |
| *My coach is less friendly to me when we do not share the same vision on certain assignments.*  Mijn coach is minder vriendelijk tegen mij als ik bepaalde opdrachten niet zie op zijn manier. |
| *My coach makes me feel guilty when I do not meet his/her expectations.*  Mijn coach zorgt er vaak voor dat ik me schuldig voel als ik zijn verwachtingen niet kon inlossen. |

**Weekly Questionnaire**

**Game circumstances**

| *Last game I was a starting player / a substitute..*  Afgelopen wedstrijd mocht ik starten / invallen. |
| --- |
| *Last game our team won / lost.*  Afgelopen wedstrijd hebben we gewonnen / verloren. |

**Perceived jusctice**

**During the past week:**

**Gedurende de afgelopen week:**

| *My coach rewarded me with a fair amount of playing time taking into account my contribution to the team*.  heeft mijn coach mij beloond met speelminuten rekening houdend met mijn bijdrage naar de ploeg toe. |
| --- |
| *My coach's choice to let me play or not play reflected my performance and commitment during training.*  De keuze van de coach om mij te laten spelen of niet-spelen reflecteerde mijn prestaties en inzet op training. |
| *My coach rewarded me with playing time during the game based on my volleyball skills.*  Mijn coach heeft mij omwille van mijn volleybalcapaciteiten – en mogelijkheden speelkansen gegeven tijdens de wedstrijd. |
| *My coach based his selection of the starting team on the talent and competence of the players*  Mijn coach baseerde zich op individueel talent en competentie voor de selectie van zijn basisteam. |
| *Every player was treated equally and no player was privileged.*  was ieder individu gelijk voor de wet en kon geen enkele speelster zich meer permitteren. |
| *My coach treated everyone on an equal basis.*  Mijn coach behandelde iedereen op gelijke basis. |
| *My individual approach by the coach was based on accurate information.*  Mijn individuele aanpak door de coach was gebaseerd op accurate informatie. |
| *The evaluation of my performance during the game and/or training was supported by reliable information.*  De evaluatie van mijn prestatie tijdens de wedstrijd en/of training werd gestaafd met betrouwbare gegevens. |
| *The manner in which the coach approached me during the training and/or game was influenced by other players.*  Mijn coach werd beïnvloed door andere speelsters in de manier waarop hij mij aanpakte tijdens de training en/of wedstrijd. |
| *My coach substituted players based on objective and accurate information (e.g., scouting data).*  Mijn coach wisselde de speelsters op basis van objectieve en nauwkeurige informatie (e.g., scoutinggegevens). |
| *My coach consequently substituted players when they were underperforming.*  Mijn coach was consequent en voerde een vervanging door als een speelster onder haar niveau presteerde. |
| After the game, my coach was willing to admit a wrong decision during the game.  Mijn coach was bereid een foute beslissing tijdens de wedstrijd achteraf toe te geven. |

**Decision justifications**

**During past week:**

**Gedurende afgelopen week**

| *I could understand the tactical guidelines and interventions of the coach.*  Kon ik de speltactische richtlijnen en ingrepen van mijn coach begrijpen. |
| --- |
| *The choice of the starting team and substitutes was clear.*  Was de spelerskeuze van mijn coach duidelijk. |
| *My coach motivated and argued his tactical decisions.*  Motiveerde en argumenteerde mijn coach zijn tactische beslissingen. |
| *I gained insight into the underlying reasons of my coach's tactical options.*  Kreeg ik inzicht in de achterliggende redenen van de tactische opties van mijn coach. |
